# Complex GABA_B_ receptor complexes: how to generate multiple functionally distinct units from a single receptor

**DOI:** 10.3389/fphar.2014.00012

**Published:** 2014-02-11

**Authors:** Chanjuan Xu, Wenhua Zhang, Philippe Rondard, Jean-Philippe Pin, Jianfeng Liu

**Affiliations:** ^1^Cellular Signaling Laboratory, Key Laboratory of Molecular Biophysics of Ministry of Education, College of Life Science and Technology, Huazhong University of Science and TechnologyWuhan, China; ^2^Institut de Génomique Fonctionnelle, CNRS UMR5203, INSERM U661, Universités de Montpellier I & IIMontpellier, France

**Keywords:** GABA_**B**_ receptor, dimers, large oligomers, G-protein coupled receptor interacting proteins, signal transduction

## Abstract

The main inhibitory neurotransmitter, GABA, acts on both ligand-gated and G protein-coupled receptors, the GABA_A/C_ and GABA_B_ receptors, respectively. The later play important roles in modulating many synapses, both at the pre- and post-synaptic levels, and are then still considered as interesting targets to treat a number of brain diseases, including addiction. For many years, several subtypes of GABA_B_ receptors were expected, but cloning revealed only two genes that work in concert to generate a single type of GABA_B_ receptor composed of two subunits. Here we will show that the signaling complexity of this unit receptor type can be largely increased through various ways, including receptor stoichiometry, subunit isoforms, cell-surface expression and localization, crosstalk with other receptors, or interacting proteins. These recent data revealed how complexity of a receptor unit can be increased, observation that certainly are not unique to the GABA_B_ receptor.

## INTRODUCTION

Neurons communicate with others to form network in the brain through the release and detection of neurotransmitters. Many receptors participate in the detection of neurotransmitters including ionotropic receptors which mediate fast responses and metabotropic receptors which induce slow and long-term plasticity regulations. Metabotropic glutamate receptors (mGluRs), which are activated by glutamate, the major excitatory neurotransmitter of the central nervous system (CNS), consist of eight subtypes named from mGluR1 to mGluR8 show different localization and signaling in synapse (Kniazeff et al., 2011). Other receptors such as serotonin receptors and dopamine receptors, which are activated by serotonin or dopamine, also have several variants (Lee et al., 2000). However, as the main inhibitory neurotransmitter in the CNS, gamma aminobutyric acid (GABA) has only one metabotropic receptor subtype, GABA_ B_ receptor (Kaupmann et al., 1998; Benke et al., 1999; Marshall et al., 1999). Located both pre-synaptically and post-synaptically, GABA_ B_ receptor is thought to play a role in CNS disorders such as epilepsy, spasticity, schizophrenia, anxiety, depression, cognitive deficits, and addiction (Bettler et al., 2004). It is also shown to be involved in cell survival, nerve growth cone guidance, migration and position of neurons (Xiang et al., 2002; McClellan et al., 2008; Zhou et al., 2008). How one GABA_ B_ receptor induces multiple downstream functions remains to be discussed. Here, we show how multiple functions can be generated from a single GABA_ B_ receptor through: (1) the oligomeric state in dimers or larger complexes; (2) subunit and isoform variants; (3) cell surface expression and localization; (4) crosstalk with other receptors GABA_ B_ receptor interacting proteins.

## DIMERIZATION AND LARGE OLIGOMIZATION OF GABA_**B**_ RECEPTOR

**FIGURE 1:**
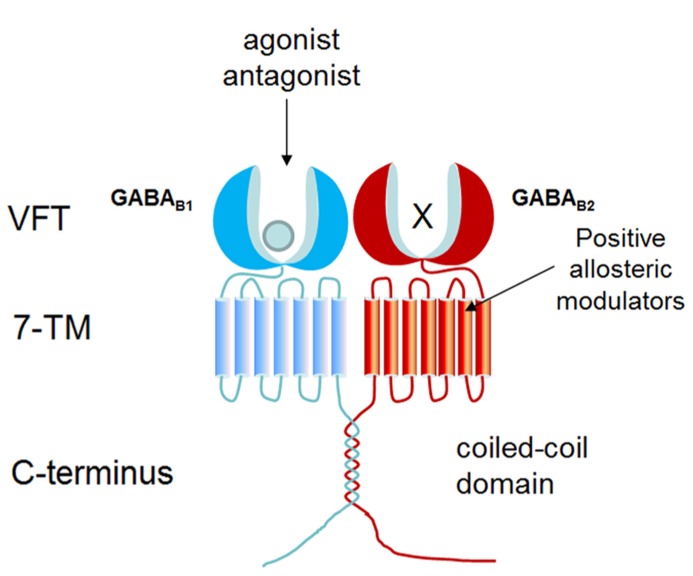
**Structure organization of GABA_**B**_ receptor.** GABA_ B_ receptor forms heterodimer composed by GABA_ B1_ and GABA_ B2_. GABA_ B1_ is responsible for ligand binding in N-terminal VFT domain, whereas the VFT of GABA_ B2_ fails to bind any known ligand. PAMs bind to GABA_ B2_ transmembrane domain to potentiate the effect of agonist.

As a member of GPCR class C, GABA_ B_ receptor consists of two subunits: GABA_ B1_ and GABA_ B2_, and functions as a heterodimer (Kaupmann et al., 1998; Marshall et al., 1999). As shown in **Figure 1**, each subunit is composed of a large extracellular domain (Venus flytrap, VFT), a seven-transmembrane domain, and an intracellular C-terminal. Although GABA_ B2_ shares 54% similarity with GABA_ B1_, only the VFT domain of GABA_ B1_ can bind ligands (such as GABA and baclofen) and orthosteric antagonists (such as CGP54626, CGP64213; Pin et al., 2004; Geng et al., 2012; Geng et al., 2013). Due to the presence of endoplasmic reticulum (ER) retention sequence (RSRR) in the C-terminal, GABA_ B1_ cannot reach the plasma membrane by itself. GABA_ B2_ masks GABA_ B1_ ER retention sequence via a coiled-coil domain to escort GABA_ B1_ to the cell surface (Galvez et al., 2001). GABA_ B2_ ectodomain does not bind GABA, but interacts with the GABA_ B1_ ectodomain to increase agonist affinity by stabilizing the agonist-bound conformation of GABA_B1 _(Liu et al., 2004; Rondard et al., 2008; Geng et al., 2012). GABA_ B2_ is also responsible for G protein coupling (Duthey et al., 2002; Havlickova et al., 2002). Following activation of G_i/o_ protein, G_α__i/o_ subunits inhibit adenylyl cyclase to reduce cAMP levels while G_β__γ_ subunits inhibit Ca^2^^+^ channels and activate K^+^ channels (Bowery et al., 2002; Ulrich and Bettler, 2007; Chalifoux and Carter, 2011).

Up to now, baclofen is the only drug targeting GABA_ B_ receptor in the market, which is used as a muscle relaxant to treat spasticity (Froestl, 2010). Positive allosteric modulators (PAMs), such as CGP7930 and GS39783, bind within GABA_ B2_ transmembrane domain to strengthen the effect of agonists (Urwyler et al., 2001). CGP7930 acts as a PAM and partial agonist through GABA_ B2_ which can facilitate agonist response at low concentration and activate the receptor alone at higher concentration (Urwyler et al., 2001; Onali et al., 2003; Binet et al., 2004; Tu et al., 2007).

Dimers, tetramers, or higher order oligomers of GABA_ B_ receptor can be detected both in heterologous system (Maurel et al., 2008; Calebiro et al., 2013) and in native neurons (Schwenk et al., 2010; Comps-Agrar et al., 2011). GABA_ B_ receptor is present in equilibrium between heterodimers and higher-order oligomers, with a relative preference for tetramers (dimers of dimers) and octamers (tetramers of dimers; Calebiro et al., 2013). Whereas GABA_ B_ receptor heterodimers are stable due to strong non-covalent interactions, the higher-order oligomers are the result of weaker and likely transient interactions among heterodimers (Calebiro et al., 2013). Although agonist stimulation did not alter receptor di-/oligomerization (Calebiro et al., 2013), destabilizing the oligomers by a competitor or a GABA_ B1_ mutant revealed different G protein coupling efficiencies depending on the oligomeric state of the receptor (Comps-Agrar et al., 2012), suggesting a negative functional cooperativity between the GABA_ B_ receptor heterodimers within the large oligomers.

## GABA_**B**_ RECEPTOR SUBUNITS AND ISOFORMS

The GABA_ B_ receptor subunits GABA_ B1_ and GABA_ B2_ are co-expressed throughout the brain (Bettler et al., 2004; Lujan et al., 2004; Bettler and Tiao, 2006). GABA_ B1_ knock-out mice displayed seizures, hyperalgesia, hyperlocomotion, memory impairment, anxiety, and immobility decrease (Schuler et al., 2001; Ruttimann et al., 2004; Catalano et al., 2005) while inactivation of the GABA_ B2_ induced similar phenotype (Mombereau et al., 2005). Both the GABA_ B1_ and GABA_ B2_ subunits are essential for normal function of GABA_ B_ receptor. However, baclofen was able to inhibit K^+^ channels in the CA1 pyramidal neurons of GABA_ B2_^-^^/^^-^ mice (Gassmann et al., 2004), suggesting specific properties of GABA_ B1_ in the absence of GABA_ B2_. Mutation of the GABA_ B1_ ER retention sequence RSRR to ASAR allows GABA_ B1_ to reach the cell surface by itself (Couve et al., 1998). GABA_ B_ receptor agonist, baclofen, could induce ERK phosphorylation in cerebellar granule cells and HEK cells overexpressed GABA_ B1_ and GABA_ B2_ (Tu et al., 2007). The GABA_ B1_-ASAR mutant could also increase ERK phosphorylation through G_β__γ_ in the absence of GABA_ B2_ (Baloucoune et al., 2012). Although only GABA_ B2_ was reported to be important for G_i/o_ coupling, G_β__γ_ were found to pre-couple to C-terminal of GABA_ B_ for presynaptic inhibition (Laviv et al., 2011). These observations suggested the direct coupling from GABA_ B1_ to G protein signaling. On the other hand, GABA_ B2_ alone co-precipitated and co-expressed with M_2_ muscarinic receptor (M_2_R) in cortical neurons. Co-expression of the GABA_ B2_ rescued internalization of M_2_R and desensitization of GIRK channels induced by chronic stimulation (Boyer et al., 2009). Since GABA_ B1_ and GABA_ B2_ do not always exhibit the same expression pattern (Bettler and Tiao, 2006), the interaction between M_2_R and the GABA_ B2_ provides a possible mechanism for signaling induced by GABA_ B2_ alone. Overall, though it is well accepted GABA_ B1_ and GABA_ B2_ form a functional receptor together, it is still possible that each subunit plays individual roles, no matter when they act in heterodimer or alone.

Furthermore, 14 isoforms of the GABA_ B1_ can be generated by differential transcription or splicing of the mRNA named from GABA_ B1a_ to GABA_ B1n_ (Bettler et al., 2004). GABA_ B1a_ and GABA_ B1b_ are the most abundant isoforms expressed in brain (Benke et al., 1999). GABA_ B1c_ has a single sushi-domain and widely expressed in brain and form functional receptors in HEK cells co-expressed with GABA_ B2_ (Pfaff et al., 1999). GABA_ B1e__ /g/h/i/__ j__/__ l__ /m__ /n_ do not have transmembrane domains. Been secreted, GABA_ B1e_ strongly interacted with GABA_ B2_ and disturbed normal GABA_ B1_/GABA_ B2_ association, but failed to disrupt G-protein coupled inwardly rectifying potassium activation (Schwarz et al., 2000). Purified sushi domains of GABA_ B1j_ could impair the inhibitory effect of GABA_ B_ heteroreceptors on evoked and spontaneous glutamate release (Tiao et al., 2008). GABA_ B1g/h/i_ show similar sequence with GABA_ B1j_ containing the sushi domains followed by a unique C-terminal sequence (Jiang et al., 2012), but the function remains to be detected. The inhibitory effect of GABA_ B_ heteroreceptors-induced potassium current was also found in GABA_ B1l_ and GABA_ B1m_, but not for GABA_ B1k_ (Lee et al., 2010). Other isoforms like GABA_ B1d/f_ were mostly detected in transcription expression profile and no function was confirmed yet (Jiang et al., 2012). GABA_B1a_ and GABA_ B1b_ are well studied compared with others. GABA_ B1a_ has two additional sushi domains in the N-terminal region, compared with GABA_ B1b_ (Blein et al., 2004). Due to the presence of these sushi domains, GABA_ B1a_ preferentially targets to the axon terminals of excitatory synapses. Post-synaptically, both isoforms were found in dendrites, but only GABA_ B1b_ could localize in spine heads (Vigot et al., 2006; Biermann et al., 2010). GABA_ B1b_ was responsible for mediating postsynaptic inhibition of Ca^2^^+^ spikes, whereas presynaptic inhibition of GABA release was mediated by GABA_ B1a_ (Perez-Garci et al., 2006). GABA_ B1a_, but not GABA_ B1b_, was involved in impaired synaptic plasticity in hippocampus long-term potentiation (Vigot et al., 2006), emphasizing the molecular differences in synaptic GABA_ B_ functions. GABA_ B1a_ and GABA_ B1b_ also contributed differentially to GABA_ B_ receptor-mediated cognitive processes such as spontaneous alternation, object recognition and passive avoidance (Jacobson et al., 2007). Till now, no difference has been shown on molecular pharmacology between GABA_ B1a_ and GABA_ B1b_ (Billinton et al., 2001). However, CHOP was found to subtype-selective interact with GABA_ B1a_ but not GABA_ B1b_ and reduced GABA_ B__ 1a_/GABA_ B2_ receptor cell surface expression (Sauter et al., 2005), suggesting the functional diversity mediated by GABA_ B1a_ and GABA_ B1b_ through different protein-protein interactions.

## GABA_B_ RECEPTOR CELL SURFACE EXPRESSION AND LOCALIZATION

Control of cell surface GABA_ B_ receptor expression plays an important role in the regulation of receptor efficacy. GABA_ B_ receptor cell surface expression is remarkably stable and baclofen treatment did not elicit conventional β-arrestin recruitment (Couve et al., 2002; Fairfax et al., 2004). However, GABA_ B_ receptor undergoes rapid constitutive receptor internalization (Grampp et al., 2007). The balance between sorting and degradation after internalization and rapid recycling process maintains its cell surface expression stability (Grampp et al., 2008). Phosphorylation of serine892 in the C-terminus of GABA_ B2_ is important for cell surface expression stability. Chronic agonist stimulation de-phosphorylates serine892 in GABA_ B2_ and decreases GABA_ B_ receptor cell surface expression (Couve et al., 2002; Fairfax et al., 2004). The interaction between endogenous protein Mupp1 and GABA_ B2_ also plays a role to maintain GABA_ B_ receptor membrane stability (Balasubramanian et al., 2007).

Lipid rafts are specialized microdomains compartmentalize cellular processes by serving as organizing centers for the assembly of signaling molecules to regulate signal transduction. The GABA_ B_ receptor and its downstream effectors, G_α__ i_ and G_α__ o_ proteins, are all localized in lipid rafts (Becher et al., 2001, 2004). Interestingly, GABA_ B_ receptors exhibited a lower GTPγS response to agonist binding in raft-enriched fractions than in whole membranes (Becher et al., 2004), suggesting that changes in membrane environment may regulate its function. Activation of 5-HT_ 1a_ receptor could target it to lipid rafts and facilitated receptor-mediated signal transduction (Renner et al., 2007), whereas mu-opioid receptor agonists promoted receptor exiting from lipid rafts (Zheng et al., 2008). Examination of the dynamic lateral diffusion of GABA_ B_ receptors at the cell surface revealed restricted mobility of GABA_ B2_. After activation by baclofen, levels of the mobile fraction were significantly increased (Pooler and McIlhinney, 2007). Furthermore, by using single-molecule analysis of fluorescently labeled GPCR revealed that larger oligomers of GABA_ B_ receptor were prevalently organized into ordered arrays (Calebiro et al., 2013). Agonist stimulation increased the mobility of large oligomer of GABA_ B_ receptor on the cell surface (Calebiro et al., 2013). These data suggested the possibility of GABA_ B_ receptor mobility between lipid raft and non-lipid raft domains. Given that the level of cell surface GABA_ B_ receptors is highly stable following activation, lateral diffusion of GABA_ B_ receptor might provide another mechanism for controlling its signal strength.

## CROSSTALK OF GABA_**B**_ RECEPTOR WITH OTHER RECEPTORS

### GABA_**B**_ AND GABA_**A**_ RECEPTORS

Both receptors are located pre-synaptically and post-synaptically. GABA_ A_ receptors are Cl^-^ ion channels which produce fast electrical signals, whereas GABA_ B_ receptor induced long-term modulation through G protein-regulated gene transcription and protein synthesis (Luscher et al., 2011). Crosstalk between them was identified in several cell types. Due to variants of GABA_ A_ receptor subtype in different neurons, GABA_ B_ receptor showed multiple functions. In developing hypothalamic neurons, GABA_ B_ receptor activation can depress GABA_ A_ receptor-mediated Ca^2^^+^ rise by both reducing the synaptic release of GABA presynaptically and decreasing the postsynaptic Ca^2^^+^ responsiveness (Obrietan and van den Pol, 1998). In dentate gyrus granule cells, GABA_ B_ receptors showed remarkable distribution overlap with GABA_ A_ receptor on post-synaptic dendritic and somatic membranes. GABA_ B_ receptors enhanced tonic inhibition induced by extrasynaptic GABA_ A_ receptor (Tao et al., 2013). This was also observed in ventrobasal thalamus and cerebellar granule cells, but absent in CA1 pyramidal cells or layer 2/3 cortical pyramidal neurons (Connelly et al., 2013; Tao et al., 2013). One explanation is that postsynaptic GABA_ B_ receptor is possible to preferentially modulate δ–type subunit containing GABA_ A_ receptor which is dominant in dentate gyrus granule cells, compared with α5–type subunit containing GABA_ A_ receptor, which is expressed in CA1 pyramidal cells (Caraiscos et al., 2004; Glykys et al., 2008). The γ2 subunit of GABA_ A_ was found to interact with GABA_ B_ receptors and regulate GABA_ B_ receptor internalization (Balasubramanian et al., 2004). On the other hand, activation of GABA_ B_ receptor promotes GABA_ A_ receptor cell surface expression through increasing secreting brain-derived neurotrophic factor (BDNF) and PLC/DAG/PKC activation (Kuczewski et al., 2011). The crosstalk between GABA_ B_ and GABA_ A_ receptors shows possibility for drug co-application in disease treatment. In animal model, co-application of both of their agonists: muscimol and baclofen protected hippocampal CA1 neurons in cerebral ischemic injury (Zhang et al., 2007). Tiagabine and vigabatrin which increase GABA level in the brain and affect both GABA_ B_ and GABA_ A_ receptor activity, are effective in treating alcohol addiction (Tyacke et al., 2010).

### GABA_**B**_ RECEPTORS AND mGluR1s

Metabotropic glutamate receptors 1 also belongs to GPCR class C as GABA_ B_ receptor. It is coupled to G_q_ protein to increase IP3 production and Ca^2^^+^ flux when activated by glutamate (Mao et al., 2005). Both receptors exhibited a high co-localization in the dendritic spine of Purkinje cells (Kamikubo et al., 2007; Rives et al., 2009) and co-immunoprecipitated from brain lysates (Tabata et al., 2004), but no oligomerization of GABA_ B_ receptor and mGluR1a was observed (Rives et al., 2009), suggesting the existence of a GABA_ B_-mGluR1 receptor complex but no direct physical contact. GABA_ B_ receptor enhanced the long-term depression of a glutamate-evoked current and increased the magnitude of depression in cerebellar parallel fiber–Purkinje cell synapses (Kamikubo et al., 2007). PLC, G_α__i/o_ and G_β__γ_ subunits was involved in GABA_ B_ receptor potentiated mGluR1 signaling (Rives et al., 2009). Baclofen regulated mGluR1-current was concentration dependent: a low concentration of baclofen showed augment effect while higher concentration showed inhibition (Hirono et al., 2001). The crosstalk was also related to mGluR1a receptor expression: more potentiation by GABA_ B_ receptor when mGluR1a receptors were less expressed (Rives et al., 2009). It suggests a precise control of two receptors for the balance of neuronal inhibition and activation. The crosstalk between G_ i/o_-coupled and G_ q_-coupled receptors which is independent of direct interaction was also observed in other receptors such as mGluR1a and mGluR2 or GABA_ B_ and 5-HT_ 2c_ receptor (Rives et al., 2009). The co-compartmentalization of these receptors and other scaffold proteins like G proteins, homer to assemble in platforms ensures the crosstalk specificity (Bockaert et al., 2004).

### GABA_**B**_ AND NMDA RECEPTORS

Gamma aminobutyric acid(B) receptor cell surface expression is independent of agonist stimulation but controlled by glutamate. Application of glutamate, mainly through NMDA receptor, decreased GABA_ B_ receptor cell surface expression and GABA_ B_ receptor activated K^+^ channel current (Guetg et al., 2010; Maier et al., 2010; Terunuma et al., 2010). Activated by NMDA receptors, CaMKII can directly interact with and phosphorylates serine867 in the C-terminus of GABA_ B1_ to trigger GABA_ B_ receptor endocytosis (Guetg et al., 2010). CaMKII might be a key signal molecular to modulate the crosstalk between GABA_ B_ receptor signaling and glutamate signaling as CaMKII was shown to interact with the NMDA receptor and regulate NMDA receptor controlled plasticity (Bayer et al., 2001; El Gaamouch et al., 2012). Upon NMDA receptor activation, the phosphorylation of serine783 in GABA_ B2_ was increased by AMP-dependent protein kinase (Terunuma et al., 2010). Furthermore, both presynaptic and postsynaptic GABA_ B_ receptor can regulate NMDA-mediated excitatory currents (Morrisett et al., 1991; Sun et al., 2006). Baclofen improved NMDA hypofunction-related social function and spatial memory deficient in knockout mice model (Gandal et al., 2012), suggesting the crosstalk between GABA_ B_ and NMDA receptors in two directions.

### GABA_**B**_ AND TYROSINE KINASE RECEPTORS

The transactivation of GPCR to RTK is an important signaling pathway which contributes to growth promotion activity (Delcourt et al., 2007). GABA_ B_ receptor could trigger secretion of BDNF and subsequent activation of the BDNF-related kinase TrkB receptor signaling pathway to promote the development of GABAergic synapses which is called ligand-dependent transactivation (Fiorentino et al., 2009). GABA_ B_ receptor could transactivate insulin-like growth factor-1 (IGF-1) receptor to induce Akt phosphorylation and protect cerebellar granule cells from apoptosis. This was independent of ligand IGF-1 (Tu et al., 2010). The first mechanism leads to the RTK activation in cells surrounding the activated GPCR due to a diffusion of the ligand, whereas the second mechanism is mediated by intracellular event that is limited to the signaling protein complex dynamically regulated upon receptor activation. G_i/o_ proteins were found to be pre-associated with the GABA_ B_ receptor. Upon activation, the G_α__i/o_ and G_β__γ_ subunits were released from GABA_ B_ receptor, followed by recruitment of FAK1, IGF-1 receptor, and Akt to GABA_ B_ receptor. FAK1 played a key role in coordinating this dynamic process. This dynamic of the GABA_ B_ receptor-associated complex is critical for signaling transduction and transactivation-dependent neuronal survival (Lin et al., 2012). Other RTKs such as epidermal growth factor receptor, neurotrophin receptor, platelet derived growth factor receptor, and fibroblast growth factor receptor have been investigated for other GPCRs (Peavy et al., 2001; Shah and Catt, 2004). Whether GABA_ B_ receptor transactivates other RTKs remains to be identified.

## GABA_**B**_ RECEPTOR INTERACTING PROTEINS

The intracellular GABA_ B_ receptor interacting proteins are involved in GABA_ B_ receptor functions such as cell surface expression (e.g., CHOP, MUPP1) (Sauter et al., 2005; Balasubramanian et al., 2007), desensitization (e.g., GRK4, NSF) (Perroy et al., 2003; Pontier et al., 2006) and signaling transduction (e.g., G proteins, ATF4, FAK1) (Vernon et al., 2001; Lin et al., 2012). Several new proteins have been identified recently to modulate GABA_ B_ receptor heterodimer function. 14-3-3ς interacting with GABA_ B1_ coiled-coil domain can partially bind to GABA_ B1_ coiled-coil domain and disrupt association of GABA_ B1_/GABA_ B2_ heterodimers (Couve et al., 2001). Disruption of 14-3-3ς/GABA_ B1_ interaction provides a strategy to enhance the effect of antinociceptive drugs (Laffray et al., 2012). The potassium channel tetramerization domain-containing (KCTD) protein family members KCTD8, KCTD12, KCTD12b, and KCTD16 are tightly associated with the C-terminus of GABA_ B2_ as auxiliary subunits in tetramers (Bartoi et al., 2010; Schwenk et al., 2010). This co-assembly changes the properties of the GABA_ B1_ and GABA_ B2_ core receptor in a KCTD subtype-specific manner. KCTD16 and KCTD8 led to persistent inhibition of Ca_ v_ channels activity, whereas KCTD12 and KCTD12b receptors transiently decreased Ca_ v_ channels activity (Schwenk et al., 2010; Seddik et al., 2012). Except regulating agonist potency and kinetics, KCTD12 reduced constitutive receptor internalization to increase the magnitude of receptor signaling (Ivankova et al., 2013). The expression levels of individual KCTD transcripts vary during postnatal brain development. KCTD12 and KCTD16 are widely expressed in most neurons whereas KCTD8 and KCTD12b show a restricted expression pattern (Metz et al., 2011). The distinct spatial and temporal KCTD distribution patterns might underlie functional differences in native GABA_ B_ receptor responses.

## CONCLUSION

**FIGURE 2:**
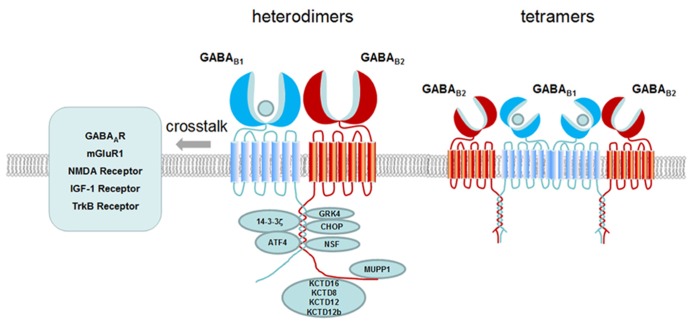
**Schematic presentation of GABA_**B**_ receptor interacting proteins and crosstalk of GABA_**B**_ receptor with other receptors.** GABA_ B_ receptor interacting proteins are binding to the C-terminus of GABA_ B_ receptor modulating the receptor membrane expression (e.g., CHOP, MUPP1), desensitization (e.g., GRK4, NSF), signaling transduction (e.g., G proteins, ATF4) and diverse function (e.g., 14-3-3ς, KCTD8, KCTD12, KCTD12b, and KCTD16). At the membrane, GABA_ B_ receptors have crosstalk with other receptors such as GABA_ A_ receptor, mGluR1, NMDA receptor, TrkB receptor and IGF-1 receptor. Both heterodimers and tetramers of GABA_ B_ receptors exist at the membrane. Dimers and dimers of GABA_ B_ receptors form tetramers through GABA_ B1_ and GABA_ B1_ interaction.

In all, we summarize how one single GABA_ B_ receptor generates multiple functions through the following aspects: (1) the composition of tetramer and large oligomer increased the complexity of the receptor; (2) Variants of subunits and isoforms contribute to functional diversity. Differential compartmentalization of the receptor variants participate in distinct function; (3) Cell surface expression and localization in lipid raft are involved in regulating receptor signaling efficacy; (4) Novel functions are generated through crosstalk with interacting proteins, auxiliary subunits or other membrane receptors as shown in **Figure 2**. The observation of the complexity generated from a single GPCR such as GABA_ B_ receptor will provide new strategy for drug development.

## Conflict of Interest Statement

The authors declare that the research was conducted in the absence of any commercial or financial relationships that could be construed as a potential conflict of interest.
